# Impact of prior immune checkpoint inhibitor on trastuzumab deruxtecan in HER2-positive advanced gastric cancer: exploratory analysis of the EN-DEAVOR study

**DOI:** 10.1093/jjco/hyaf216

**Published:** 2026-03-06

**Authors:** Yukiya Narita, Hisato Kawakami, Koki Nakanishi, Akitaka Makiyama, Naotoshi Sugimoto, Hirotaka Konishi, Satoshi Morita, Keiko Minashi, Motohiro Imano, Rin Inamoto, Tomohiro Nishina, Takeshi Kawakami, Motohisa Hagiwara, Yasuhiro Kodera, Hiroki Kume, Keita Yamaguchi, Wataru Hashimoto, Kei Muro

**Affiliations:** Department of Clinical Oncology, Aichi Cancer Center Hospital, 1-1 Kanokoden, Chikusa Ward, Nagoya, Aichi 464-8681, Japan; Department of Clinical Oncology, Tohoku University Graduate School of Medicine, 2-1 Seiryomachi, Aoba Ward, Sendai, Miyagi 980-0872, Japan; Department of Gastroenterological Surgery, Nagoya University Graduate School of Medicine, 65 Tsurumai-cho, Showa-ku, Nagoya 466-8560, Japan; Cancer Center, Gifu University Hospital, 1-1 Yanagido, Gifu 501-1112, Japan; Department of Genetic Oncology, Osaka International Cancer Institute, 3-1-69 Otemae, Chuo-ku, Osaka 540-0008, Japan; Division of Digestive Surgery, Kyoto Prefectural University of Medicine, 465 Kajiicho, Kamigyo Ward, Kyoto 602-8566, Japan; Department of Biomedical Statistics and Bioinformatics, Kyoto University Graduate School of Medicine, Yoshidakonoecho, Sakyo Ward, Kyoto 606-8303, Japan; Division of Gastroenterology, Chiba Cancer Center, 666-2 Nitonacho, Chuo Ward, Chiba 260-8717, Japan; Department of Surgery, Kindai University Faculty of Medicine, 377-2 Onohigashi, Osakasayama, Osaka 589-0014, Japan; Department of Gastroenterology, Saitama Cancer Center, 780 Komuro, Ina, Kitaadachi District, Saitama 362-0806, Japan; Department of Gastrointestinal Medical Oncology, NHO Shikoku Cancer Center, 160 Minamiumemotomachi, Matsuyama, Ehime 791-0245, Japan; Division of Gastrointestinal Oncology, Shizuoka Cancer Center, 1007 Shimonagakubo, Nagaizumi, Sunto-gun, Shizuoka 411-8777, Japan; Department of Surgery, Nihonkai General Hospital, 30 Akihocho, Sakata, Yamagata 998-8501, Japan; NHO Nagoya Medical Center, 4-1-1 Sannomaru, Naka Ward, Nagoya, Aichi 460-0001, Japan; Oncology Medical Science Department I, Daiichi Sankyo Co. Ltd., 3-5-1 Nihonbashihoncho, Chuo City, Tokyo 103-0023, Japan; Oncology Medical Science Department I, Daiichi Sankyo Co. Ltd., 3-5-1 Nihonbashihoncho, Chuo City, Tokyo 103-0023, Japan; Data Intelligence Department, Daiichi Sankyo Co. Ltd., 1-2-58, Hiromachi, Shinagawa City, Tokyo 140-8710, Japan; Department of Clinical Oncology, Aichi Cancer Center Hospital, 1-1 Kanokoden, Chikusa Ward, Nagoya, Aichi 464-8681, Japan

**Keywords:** EN-DEAVOR, HER2-positive gastric cancer, treatment strategy, trastuzumab deruxtecan, nivolumab

## Abstract

**Background:**

Human epidermal growth factor receptor 2 (HER2)-positive advanced gastric cancer (AGC) presents significant therapeutic challenges due to its molecular heterogeneity. Previous studies suggest that immune checkpoint inhibitors (ICIs) may enhance the efficacy of subsequent HER2-targeted therapy. However, evidence suggesting an optimal sequence for nivolumab and trastuzumab deruxtecan (T-DXd) treatment is limited. This exploratory analysis of EN-DEAVOR evaluated the effectiveness and safety of administering T-DXd relative to the timing of prior ICI administration.

**Methods:**

This study assessed real-world outcomes of T-DXd in patients with HER2-positive AGC stratified by prior ICI exposure: within 2 months of nivolumab (Group A), >2 months (Group B), and no prior nivolumab (Group C). The primary effectiveness endpoints included real-world progression-free survival (rwPFS) and objective response rate (ORR). Safety endpoints included grade ≥ 3 adverse events (AEs).

**Results:**

Among 311 eligible patients, Group A showed the longest median rwPFS (n = 63; 6.9 months) compared with Group B (n = 63; 4.6 months) and Group C (n = 185; 4.2 months). The risk of progression was significantly lower in Group A compared with Group B (hazard ratio [95% confidence interval]: 0.6 [0.4–0.9]; *P* = .0074). ORR was numerically highest in Group A (54.9%) versus Group B (30.8%) and Group C (43.4%). More patients in Group B (58.7%) experienced grade ≥ 3 AEs than in Group A (50.8%) and Group C (43.8%). No new safety signals were observed.

**Conclusions:**

Initiating T-DXd within 2 months post-ICI may enhance therapeutic efficacy in HER2-positive AGC without affecting safety, supporting a potential sequencing option after ICI therapy.

## Teaser

This study showed the effectiveness of T-DXd in patients with HER2-positive gastric cancer, with optimal outcomes when initiated ≤2 months post-ICI. Grade ≥ 3 AEs were comparable across subgroups.

## Introduction

Gastric cancer is one of the leading causes of cancer-related mortality worldwide, with Japan exhibiting the highest age-standardized incidence [1]. Among patients with advanced gastric or gastroesophageal junction adenocarcinoma, ~20% exhibit overexpression of human epidermal growth factor receptor 2 (HER2), which defines a biologically and clinically distinct subgroup requiring tailored therapeutic strategies [2, 3]. For HER2-positive advanced gastric cancer (AGC), international guidelines such as the National Comprehensive Cancer Network (NCCN) and European Society for Medical Oncology (ESMO) recommend trastuzumab-based therapy in the first-line setting and trastuzumab deruxtecan (T-DXd) in subsequent lines [4, 5]. In Japan, following the results of the DESTINY-Gastric01 trial [6], T-DXd received approval in September 2020 and has since been incorporated as a third-line recommendation in the Japanese Gastric Cancer Association (JGCA) 2021 guidelines [7]. Before T-DXd became available, nivolumab, an immune checkpoint inhibitor (ICI), was the most commonly prescribed drug as third- or later-line treatment in patients with HER2-positive AGC in Japan [8].

Emerging evidence has suggested that HER2-positive tumors may exhibit an immunologically active microenvironment, including increased programmed death-ligand 1 (PD-L1) expression, and that prior ICI exposure may potentiate the effects of subsequent therapies [9]. Preclinical studies have shown that HER2 signaling plays a role in regulating the recruitment and activation of tumor-infiltrating immune cells, and anti–programmed death protein (PD)-1 antibodies could significantly increase the therapeutic activity of HER2 inhibitors [10, 11]. Iwata et al. reported that combination therapy with T-DXd and ICI resulted in enhanced antitumor immunity compared with that observed with either monotherapy [12]. Furthermore, Osa et al. also reported that nivolumab showed controlled levels of lymphocyte-surface PD-1–blocking activity for more than 2 months [13].

Although the DESTINY-Gastric01 trial included some patients with prior ICI exposure and showed numerically better outcomes in this subgroup, the impact of ICI–T-DXd sequencing and timing has not been fully characterized in the real-world setting [14]. The EN-DEAVOR study, a nationwide retrospective observational cohort study in Japan, previously confirmed the effectiveness and safety of T-DXd in routine clinical practice [15]. While previous analyses within this study reported no differences in overall survival (OS) and real-world progression-free survival (rwPFS) based on prior ICI therapy, a comprehensive evaluation of the sequencing and timing between ICI and T-DXd therapies has not yet been performed [15, 16].

In this exploratory analysis of EN-DEAVOR, we explored whether the timing of T-DXd administration in relation to prior nivolumab therapy affects clinical outcomes. We compared effectiveness and safety across subgroups stratified by the interval between the last dose of nivolumab and the first dose of T-DXd. This analysis provides real-world evidence regarding the potential synergy of subsequent T-DXd therapy in patients who previously received ICI-containing regimens.

## Patients and methods

### Study design

This exploratory post hoc subgroup analysis was conducted using the EN-DEAVOR data set. EN-DEAVOR was a retrospective, observational, real-world cohort study conducted across 63 sites in Japan between 25 September 2020 and 30 September 2021 (UMIN000049032) [15]. The study was approved by the institutional ethical review board of Nagoya University Graduate School of Medicine (No. 2022–0170). All patients provided written informed consent to participate in this study. However, opt-out registration was allowed for patients when obtaining written informed consent was challenging due to various reasons.

### Eligibility criteria and subgroups

Patients aged ≥20 years with a histopathologically confirmed diagnosis of HER2-positive (immunohistochemistry [IHC] 3+ or IHC 2+ with *in situ* hybridization [ISH]+) unresectable advanced or recurrent gastric or gastroesophageal junction adenocarcinoma that had worsened after chemotherapy and who had started treatment with T-DXd were included in the primary analysis [15]. This post hoc analysis examined the following patient subgroups: (i) those who received their first dose of T-DXd treatment within 2 months after the final dose of nivolumab (Group A), (ii) those who received their first dose of T-DXd treatment more than 2 months after the final dose of nivolumab (Group B), and (iii) those who did not receive nivolumab prior to T-DXd treatment (Group C). The cutoff of 2 months was selected based on the findings by Osa et al [13].

### Study endpoints

The primary effectiveness endpoints included rwPFS across the three patient subgroups, as well as objective response rate (ORR), disease control rate (DCR), and best overall response (BOR) in patients presenting with target lesions. Exploratory endpoints included median OS and relative OS and relative rwPFS based on different time intervals between the first dose of T-DXd administration and the final dose of nivolumab treatment (i.e. ≤2/>2 months, ≤3/>3 months, ≤4/>4 months, and ≤5/>5 months). The exploratory analysis also included types of treatments administered to participants after T-DXd. The safety endpoint included the incidence of grade ≥ 3 adverse events (AEs) in the three patient subgroups.

### Statistical analysis

The sample size was set to 300 patients considering the data collection feasibility at the participating study sites. The analysis population comprised all patients registered for the study, except those with significant protocol deviations and those deemed ineligible at the case-review meeting. For the univariable and multivariable analyses, *P* values were calculated for the Cox proportional hazards model and the logistic regression model. The significance level for hypothesis testing was 5% two-sided. No extra complementing process was conducted if missing data were detected. Data are presented as hazard ratios (HRs) with 95% confidence interval (CI) for rwPFS and OS. Statistical analyses were performed using SAS software version 9.4.

## Results

### Patient disposition

Of 312 patients enrolled in the primary analysis, 63 were in Group A, 63 were in Group B, and 185 were in Group C. One patient with missing data on the duration or timing of nivolumab administration was excluded from the analysis.

### Demographics and baseline clinical characteristics

Baseline patient characteristics were generally well balanced between the three groups (Table 1). The mean (standard deviation) age was 67.3 (9.5), 68.1 (10.3), and 67.5 (11.3) years in Groups A, B, and C, respectively. Majority of the patients across all groups were male; had an Eastern Cooperative Oncology Group Performance Status score of 0 or 1; and presented with primary lesions in the stomach, metastases in two or more organs, and with the lymph nodes as the primary site of metastasis. Group B predominantly consisted of patients (84.1%) who had undergone a higher number of prior lines of therapy (≥4). However, 66.7% of patients in Group A had received 3 prior lines of therapy, while 83.8% of patients in Group C had undergone only 1–2 prior lines of therapy, with the difference being statistically significant (*P* < .0001).

**Table 1:** Demographics and baseline clinical characteristics.

	Group A n = 63	Group B n = 63	Group C n = 185	*P* value^a^
Sex				
Male	50 (79.4)	54 (85.7)	130 (70.3)	0.4821
Age, years				
Mean [standard deviation]	67.3 [9.5]	68.1 [10.3]	67.5 [11.3]	0.6729
Median [range]	71.0 [42.0–82.0]	71.0 [34.0–83.0]	70.0 [27.0–89.0]	
ECOG PS				
0 and 1	57 (90.5)	57 (90.5)	157 (84.9)	1.000
≥2	6 (9.5)	6 (9.5)	26 (14.1)	
Unknown	0	0	2 (1.1)	
HER2 status (IHC and ISH): Before T-DXd treatment				
IHC 3+	41 (65.1)	36 (57.1)	139 (75.1)	0.0909
IHC 2+ and ISH+	21 (33.3)	20 (31.7)	44 (23.8)	
Others^b^	1 (1.6)	7 (11.1)	2 (1.1)	
Site of primary lesions				
Stomach	48 (76.2)	54 (85.7)	161 (87.0)	0.2564
Gastroesophageal junction	15 (23.8)	9 (14.3)	24 (13.0)	
Any surgeries for primary lesions				
None	41 (65.1)	37 (58.7)	126 (68.1)	0.5823
Yes	22 (34.9)	26 (41.3)	59 (31.9)	
Histological type of primary lesions				
Diffuse	12 (19.0)	17 (27.0)	50 (27.0)	0.0198
Intestinal	39 (61.9)	37 (58.7)	94 (50.8)	
Others	9 (14.3)	1 (1.6)	11 (5.9)	
Unknown	3 (4.8)	8 (12.7)	30 (16.2)	
Number of metastatic organs				
0	1 (1.6)	0 (0.0)	0 (0.0)	0.8546
1	23 (36.5)	25 (39.7)	70 (37.8)	
≥2	39 (61.9)	38 (60.3)	115 (62.2)	
Metastasis site^c^				
Lymph nodes	45 (71.4)	44 (69.8)	115 (62.2)	1.0000
Liver	35 (55.6)	40 (63.5)	84 (45.4)	0.4680
Peritoneum	15 (23.8)	16 (25.4)	84 (45.4)	1.0000
Lung	15 (23.8)	17 (27.0)	37 (20.0)	0.8381
Bones	7 (11.1)	3 (4.8)	19 (10.3)	0.3234
Brain	5 (7.9)	3 (4.8)	1 (0.5)	0.7175
Others	6 (9.5)	4 (6.3)	15 (8.1)	0.7436
Ascites				
None	41 (65.1)	45 (71.4)	86 (46.5)	0.2052
Yes	22 (34.9)	16 (25.4)	97 (52.4)	
mGPS				
0 and 1	42 (68.9)	41 (67.2)	145 (80.1)	1.0000
2	19 (31.1)	20 (32.8)	36 (19.9)	
Prior lines of therapy				
1 and 2	4 (6.3)	2 (3.2)	155 (83.8)	<0.0001
3	42 (66.7)	8 (12.7)	23 (12.4)	
≥4	17 (27.0)	53 (84.1)	7 (3.8)	
Nivolumab treatment history				
Time from the final dose of nivolumab to the first dose of T-DXd treatment (months), median [range]	0.76 [0.1–1.9]	8.77 [2.3–28.6]	-	<0.0001

Data are presented as n (%) unless otherwise specified.

Group A, patients who received their first dose of T-DXd treatment within 2 months after the final dose of nivolumab; Group B, patients who received their first dose of T-DXd treatment more than 2 months after the final dose of nivolumab; Group C, patients who did not receive nivolumab prior to T-DXd treatment.

ECOG PS, Eastern Cooperative Oncology Group Performance Status; HER2, human epidermal growth factor receptor 2; IHC, immunohistochemistry; ISH, *in situ* hybridization; mGPS, modified Glasgow prognostic score; T-DXd, trastuzumab deruxtecan.

^a^Fisher’s exact test was used for group comparisons between Group A and Group B only (excluding Group C).

^b^Cases in which HER2 positivity was confirmed, but the corresponding HER2 status information was unavailable.

^c^Multiple selections were allowed.

### Primary effectiveness endpoints

**Figure 1:**
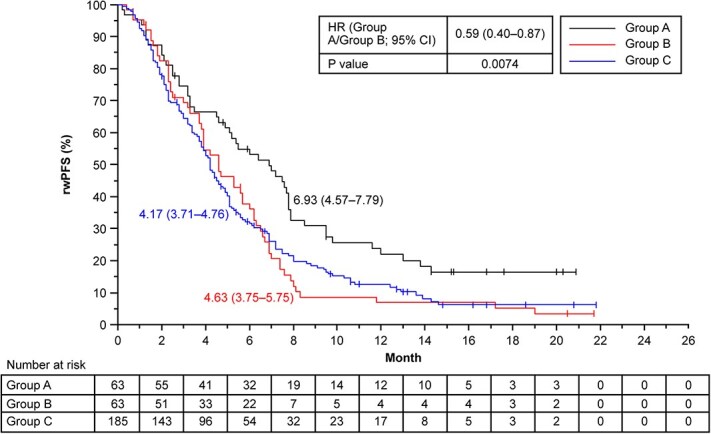
Kaplan–Meier analysis of rwPFS by timing of ICI therapy prior to T-DXd administration. The numbers alongside the curves indicate the median rwPFS and 95% CI. Group A, patients who received their first dose of T-DXd treatment within 2 months after the final dose of nivolumab; Group B, patients who received their first dose of T-DXd treatment more than 2 months after the final dose of nivolumab; Group C, patients who did not receive nivolumab prior to T-DXd treatment. CI, confidence interval; HR, hazard ratio; ICI, immune checkpoint inhibitor; rwPFS, real-world progression-free survival; T-DXd, trastuzumab deruxtecan.

The median (95% CI) rwPFS (months) was 6.93 (4.57–7.79), 4.63 (3.75–5.75), and 4.17 (3.71–4.76) in Groups A, B, and C, respectively (Fig. 1). The risk of progression was significantly lower in Group A compared with Group B (HR [95% CI]: 0.59 [0.40–0.87]; *P* = .0074; Fig. 1).

The ORR was numerically higher in Group A at 54.9% (95% CI: 40.3–68.9) compared with Group B at 30.8% (95% CI: 18.7–45.1; Table 2). The DCR was comparable across groups: 80.4% (95% CI: 66.9–90.2) in Group A, 82.7% (95% CI: 69.7–91.8) in Group B, and 82.0% (95% CI: 74.0–88.3) in Group C (Table 2). The BOR demonstrated varying outcomes among the three groups. Partial response (PR) was most notable in Group A, where 51.0% of patients achieved a tumor reduction, compared with 28.8% in Group B and 41.8% in Group C. Stable disease (SD) was observed most frequently in Group B, with 51.9% of patients showing no disease progression, compared with 25.5% and 38.5% in Groups A and C, respectively (Table 2).

**Table 2:** ORR, DCR, and BOR in patients with a target lesion (all patients).

	Group A (n = 51)	Group B (n = 52)	Group C (n = 122)
ORR, n (%)	28 (54.9)	16 (30.8)	53 (43.4)
95% CI^a^	40.3–68.9	18.7–45.1	34.5–52.7
DCR, n (%)	41 (80.4)	43 (82.7)	100 (82.0)
95% CI^a^	66.9–90.2	69.7–91.8	74.0–88.3
BOR, n (%)			
CR	2 (3.9)	1 (1.9)	2 (1.6)
PR	26 (51.0)	15 (28.8)	51 (41.8)
SD	13 (25.5)	27 (51.9)	47 (38.5)
PD	7 (13.7)	7 (13.5)	17 (13.9)
NE^b^	3 (5.9)	2 (3.8)	5 (4.1)

Group A, patients who received their first dose of T-DXd treatment within 2 months after the final dose of nivolumab; Group B, patients who received their first dose of T-DXd treatment more than 2 months after the final dose of nivolumab; Group C, patients who did not receive nivolumab prior to T-DXd treatment.

BOR, best overall response; CI, confidence interval; CR, complete response; DCR, disease control rate; NE, not evaluable; ORR, objective response rate; PD, progressive disease; PR, partial response; SD, stable disease; T-DXd, trastuzumab deruxtecan.

^a^Determined using the Clopper-Pearson method.

^b^Including non-implemented.

### Exploratory endpoints

**Figure 2:**
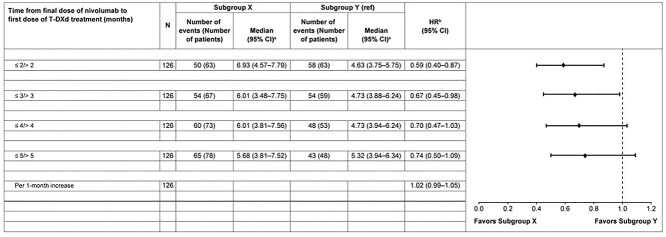
Relative rwPFS across subgroups stratified by timing of ICI therapy prior to T-DXd administration. ^a^Using the Brookmeyer and Crowley method. ^b^Using the Cox proportional hazards model. Subgroup X, patients who received their first dose of T-DXd within a shorter time interval (≤2, ≤3, ≤4, or ≤5 months) following the final dose of nivolumab; Subgroup Y, patients who initiated T-DXd after a longer time interval (>2, >3, >4, or >5 months) following the final nivolumab dose. CI, confidence interval; HR, hazard ratio; ref, reference; rwPFS, real-world progression-free survival; T-DXd, trastuzumab deruxtecan.

The median (95% CI) OS (months) was 11.56 (7.03–13.50), 8.15 (6.83–9.00), and 9.89 (7.46–11.83) in Groups A, B, and C, respectively (Fig. S1). The survival rate was numerically higher in Group A compared with Group B (HR [95% CI]: 0.79 [0.53–1.17]; *P* = .2397; Fig. S1). Overall, there was a slight trend that survival rate was higher in patients who received T-DXd treatment ≤2/>2 months after nivolumab therapy (HR [95% CI]: 0.79 [0.53–1.17]) compared with those who received T-DXd treatment ≤3/>3 months (HR [95% CI]: 0.87 [0.58–1.30]), ≤4/>4 months (HR [95% CI]: 0.97 [0.65–1.46]), and ≤5/>5 months (HR [95% CI]: 0.93 [0.62–1.40]) after nivolumab therapy (Fig. S2). Overall, risk of progression was lower in patients who received T-DXd treatment ≤2/>2 months after nivolumab therapy (HR [95% CI]: 0.59 [0.40–0.87]) compared with those who received T-DXd treatment ≤3/>3 months (HR [95% CI]: 0.67 [0.45–0.98]), ≤4/>4 months (HR [95% CI]: 0.70 [0.47–1.03]), and ≤5/>5 months (HR [95% CI]: 0.74 [0.50–1.09]) after nivolumab therapy (Fig. 2).

The treatment rates post–T-DXd therapy were 54.0%, 47.6%, and 70.3% in Groups A, B, and C, respectively (Table S1). Large proportions of patients in all the groups received chemotherapy (Group A: 50.8%; Group B: 39.7%; Group C: 65.9%).

Multivariate analysis incorporating the interval between completion of prior nivolumab therapy and initiation of T-DXd showed that patients who received T-DXd within a shorter interval after nivolumab completion experienced significantly improved rwPFS outcomes (HR [95% CI]: 0.63 [0.44–0.89]; *P* = .0081; Table S2). No significant differences were observed for OS or ORR (Tables S3  **&**  S4).

### Safety endpoints

The incidence rate of grade ≥ 3 AEs was stratified by duration of prior trastuzumab treatment (Table 3). More patients in Group B (58.7%) experienced grade ≥ 3 AEs than in Group A (50.8%) and Group C (43.8%). In Group A, hematotoxicity was observed in 25.4% of patients, with neutrophil count reduction being the most common hematologic event (22.2%). In Group B, hematotoxicity was more prominent, affecting 42.9% of patients, with anemia being the most frequent hematologic AE (27.0%). Decreased neutrophil count and anorexia occurred in 19.0% and 7.9% of patients, respectively. The incidence of interstitial pneumonia was 6.3% in both Groups A and B and 3.2% in Group C.

**Table 3:** Incidence rate of grade ≥ 3 AEs.

	Group A n = 63	Group B n = 63	Group C n = 185
AEs	32 (50.8)	37 (58.7)	81 (43.8)
Hematotoxicity	16 (25.4)	27 (42.9)	45 (24.3)
Non-hematotoxicity	20 (31.7)	22 (34.9)	46 (24.9)
Nausea	3 (4.8)	2 (3.2)	8 (4.3)
Vomiting	1 (1.6)	1 (1.6)	4 (2.2)
Anorexia	3 (4.8)	5 (7.9)	21 (11.4)
Malaise	3 (4.8)	4 (6.3)	7 (3.8)
Anemia	3 (4.8)	17 (27.0)	9 (4.9)
Diarrhea	1 (1.6)	1 (1.6)	3 (1.6)
Constipation	0 (0.0)	0 (0.0)	0 (0.0)
Fatigue	2 (3.2)	0 (0.0)	1 (0.5)
Neutrophil count decreased	14 (22.2)	12 (19.0)	35 (18.9)
Platelet count decreased	1 (1.6)	3 (4.8)	8 (4.3)
White blood cell decreased	1 (1.6)	4 (6.3)	5 (2.7)
Lymphocyte count decreased	0 (0.0)	1 (1.6)	0 (0.0)
Febrile neutropenia	1 (1.6)	2 (3.2)	1 (0.5)
Interstitial pneumonia	4 (6.3)	4 (6.3)	6 (3.2)
Pneumonitis	0 (0.0)	1 (1.6)	4 (2.2)
Infusion-related reaction	0 (0.0)	0 (0.0)	0 (0.0)
Others	9 (14.3)	7 (11.1)	11 (5.9)

Data are presented as n (%).

Group A, patients who received their first dose of T-DXd treatment within 2 months after the final dose of nivolumab; Group B, patients who received their first dose of T-DXd treatment more than 2 months after the final dose of nivolumab; Group C, patients who did not receive nivolumab prior to T-DXd treatment.

AE, adverse event; T-DXd, trastuzumab deruxtecan.

## Discussion

The previous EN-DEAVOR study assessing the real-world effectiveness of T-DXd in Japanese patients with HER2+ AGC showed no difference in efficacy regardless of prior ICI therapy, such as with nivolumab, indicating that nivolumab administration was not a prognostic factor for OS [15] or rwPFS [16]. To the best of our knowledge, this is the first real-world report to examine the detailed impact of timing between prior ICI therapy and subsequent T-DXd treatment in patients with HER2-positive AGC. This exploratory analysis of EN-DEAVOR demonstrated that initiating T-DXd within 2 months following the final dose of nivolumab was associated with significantly improved rwPFS and numerically higher ORR, compared to delayed administration. These benefits were achieved without an increase in grade ≥ 3 AEs, supporting the feasibility of this sequencing strategy in clinical practice.

One plausible explanation for the favorable rwPFS in patients who started T-DXd earlier is the persistence of immune modulation induced by prior nivolumab therapy [12]. Sustained PD-1 receptor occupancy, as demonstrated by Osa et al., may maintain an activated immune milieu during the initial phase of T-DXd treatment [13]. Given that HER2 signaling has been implicated in modulating immune cell dynamics within the tumor microenvironment, T-DXd may exert greater efficacy when administered in this preconditioned immunologic setting [9–11]. The interplay between these mechanisms suggests that treatment sequencing could be a key determinant of therapeutic success.

The findings of this study align with previous exploratory analyses of the DESTINY-Gastric-01 trial focusing on the effects of ICI, suggesting that prior immunotherapy might increase the efficacy of subsequent treatment with T-DXd [14]. Moreover, the REVIVE real-world study suggested enhanced effectiveness of chemotherapy following ICI, further supporting the hypothesis that recent immunotherapy may sensitize tumors to subsequent systemic treatments [17]. Our results extend these observations to a large real-world cohort and provide additional evidence that close sequencing of ICI and T-DXd may confer clinical benefit.

Interestingly, while rwPFS was significantly prolonged in Group A, no statistically significant difference in OS was observed between groups. Although the baseline characteristics were mostly comparable between the study groups, the majority of patients in Group A (66.7%) and Group B (84.1%) received T-DXd after 3 and ≥4 prior lines of therapy, respectively. The difference in the number of lines of therapy in each group needs to be considered carefully. Although the DESTINY-Gastric-01 trial suggested comparable T-DXd efficacy regardless of the number of prior lines of therapy, the potential impact on OS in more heavily pretreated populations remains uncertain [6]. However, no data were available regarding its impact on rwPFS. Therefore, while OS remains a clinically meaningful endpoint, it may be more susceptible to variability in treatment patterns and patient characteristics in non-randomized, observational cohorts, and should be interpreted in this setting.

From a safety perspective, the incidence of grade ≥ 3 AEs was comparable across subgroups, and no new safety signals were identified. The slightly higher hematologic toxicity observed in Group B may be attributable to more intensive prior treatment history. Notably, interstitial lung disease, a known risk of both nivolumab and T-DXd, occurred at a relatively low rate and did not appear to be exacerbated by close sequencing of the two agents [6, 17]. This supports the clinical feasibility of administering T-DXd shortly after ICI, although careful monitoring remains essential.

This study has several clinical implications. As ICIs are increasingly used in earlier lines of therapy, including as part of first-line regimens for HER2-positive AGC, our findings suggest that initiating T-DXd soon after ICI cessation may leverage the residual immunologic effects of PD-1 blockade and improve treatment outcomes. These insights may be particularly valuable in treatment settings where T-DXd is approved for second-line use and may inform the design of future sequencing strategies in HER2-positive AGC.

Several limitations must be acknowledged. First, this was a retrospective, post hoc analysis of an observational cohort, and treatment assignment was not randomized. Second, the small number of patients or the prior use of ICI therapy may have led to imbalances in key baseline characteristics, such as the number of prior lines of therapy, which can impact clinical outcomes, and the results should be interpreted with caution as T-DXd was administered immediately after prior ICI (within 2 months) more commonly in patients who had received 3 lines of therapy compared with patients who had received 4 lines of therapy. Although both ICI and T-DXd can be administered as third-line monotherapy, as stated in the Japanese guidelines [7], the treatment sequence of ICI after T-DXd has not been investigated in this study; therefore, it is not possible to ascertain whether there would be differences in treatment outcome based on treatment sequence and which treatment sequence should be followed. Third, PD-L1 combined positive score (CPS) data were not consistently available in this cohort. As CPS may influence ICI response and treatment selection, especially in the first-line setting for HER2-positive AGC, its absence limits interpretation of immunologic context and may represent a potential confounder. Finally, assessment of therapeutic effects was based on data collected in real-world clinical practice and was not based on standardized criteria. Validation of these results using a prospective clinical trial approach is needed.

## Conclusion

T-DXd was effective regardless of prior ICI treatment, but the timing of its initiation following ICI therapy appears to influence treatment outcomes. This study showed the effectiveness of T-DXd across subgroups, with optimal outcomes when initiated promptly after nivolumab therapy. Given the evolving treatment landscape for gastric cancer, including the increasing use of ICI agents in first-line therapy, the results of this study may support clinicians in determining the optimal sequencing of therapies to provide best possible outcomes for patients with AGC.

## Supplementary Material

hyaf216_Supplementary_material_ENDEAVOR_subpt_2_23Dec25

## Data sharing

The anonymized data underlying the results presented in this manuscript may be made available to researchers upon submission of a reasonable request to the corresponding author. The decision to disclose the data will be made by the corresponding author and the funder, Daiichi Sankyo Co. Ltd.
