# Supplementary material for: Impact of prior immune checkpoint inhibitor on trastuzumab deruxtecan in HER2-positive advanced gastric cancer: exploratory analysis of the EN-DEAVOR study
Source: Jpn J Clin Oncol. 2026 Mar 6;56(5):538–45. doi: 10.1093/jjco/hyaf216 (PMC13149310; doi:10.1093/jjco/hyaf216)
Supplement: hyaf216_Supplementary_material_ENDEAVOR_subpt_2_23Dec25 [file hyaf216_supplementary_material_endeavor_subpt_2_23dec25.docx]

**Supplementary material**

Impact of prior immune checkpoint inhibitor on trastuzumab deruxtecan in HER2‑positive advanced gastric cancer: exploratory analysis of the EN‑DEAVOR study

**Authors:**

Yukiya Narita^1*^, Hisato Kawakami^2^, Koki Nakanishi^3^, Akitaka Makiyama^4^, Naotoshi Sugimoto^5^, Hirotaka Konishi^6^, Satoshi Morita^7^, Keiko Minashi^8^, Motohiro Imano^9^, Rin Inamoto^10^, Tomohiro Nishina^11^, Takeshi Kawakami^12^, Motohisa Hagiwara^13^, Yasuhiro Kodera^14^, Hiroki Kume^15^, Keita Yamaguchi^15^, Wataru Hashimoto^16^, Kei Muro^1^

**Affiliations:**

^1^Department of Clinical Oncology, Aichi Cancer Center Hospital, 1-1 Kanokoden, Chikusa Ward, Nagoya, Aichi 464-8681, Japan

^2^Department of Clinical Oncology, Tohoku University Graduate School of Medicine, 2-1 Seiryomachi, Aoba Ward, Sendai, Miyagi 980-0872, Japan

^3^Department of Gastroenterological Surgery, Nagoya University Graduate School of Medicine, 65 Tsurumai-cho, Showa-ku, Nagoya 466-8560, Japan

^4^Cancer Center, Gifu University Hospital, 1-1 Yanagido, Gifu 501-1112, Japan

^5^Department of Genetic Oncology, Osaka International Cancer Institute, 3-1-69 Otemae, Chuo-ku, Osaka 540-0008, Japan

^6^Division of Digestive Surgery, Kyoto Prefectural University of Medicine, 465 Kajiicho, Kamigyo Ward, Kyoto 602-8566, Japan

^7^Department of Biomedical Statistics and Bioinformatics, Kyoto University Graduate School of Medicine, Yoshidakonoecho, Sakyo Ward, Kyoto 606-8303, Japan

^8^Division of Gastroenterology, Chiba Cancer Center, 666-2 Nitonacho, Chuo Ward, Chiba 260-8717, Japan

^9^Department of Surgery, Kindai University Faculty of Medicine, 377-2 Onohigashi, Osakasayama, Osaka 589-0014, Japan

^10^Department of Gastroenterology, Saitama Cancer Center, 780 Komuro, Ina, Kitaadachi District, Saitama 362-0806, Japan

^11^Department of Gastrointestinal Medical Oncology, NHO Shikoku Cancer Center, 160 Minamiumemotomachi, Matsuyama, Ehime 791-0245, Japan

^12^Division of Gastrointestinal Oncology, Shizuoka Cancer Center, Sunto-gun, 1007 Shimonagakubo, Nagaizumi, Sunto-gun, Shizuoka 411-8777, Japan

^13^Department of Surgery, Nihonkai General Hospital, 30 Akihocho, Sakata, Yamagata 998-8501, Japan

^14^NHO Nagoya Medical Center, 4-1-1 Sannomaru, Naka Ward, Nagoya, Aichi 460-0001, Japan

^15^Oncology Medical Science Department I, Daiichi Sankyo Co. Ltd., 3-5-1 Nihonbashihoncho, Chuo City, Tokyo 103-0023, Japan

^16^Data Intelligence Department, Daiichi Sankyo Co. Ltd., 1-2-58, Hiromachi, Shinagawa City, Tokyo 140-8710, Japan

***Corresponding author:**

Yukiya Narita, MD

Department of Clinical Oncology,

Aichi Cancer Center Hospital,

1-1 Kanokoden, Chikusa Ward,

Nagoya, Aichi 464-8681, Japan.

Telephone number: 052-762-6111.

Fax number: +81-52-764-2963.

E-mail address: [yukiya.narita@aichi-cc.jp](mailto:yukiya.narita@aichi-cc.jp)

ORCID ID: 0000-0002-8018-0077

**Figure S1** Kaplan-Meier analysis of OS by timing of ICI therapy prior to T-DXd administration


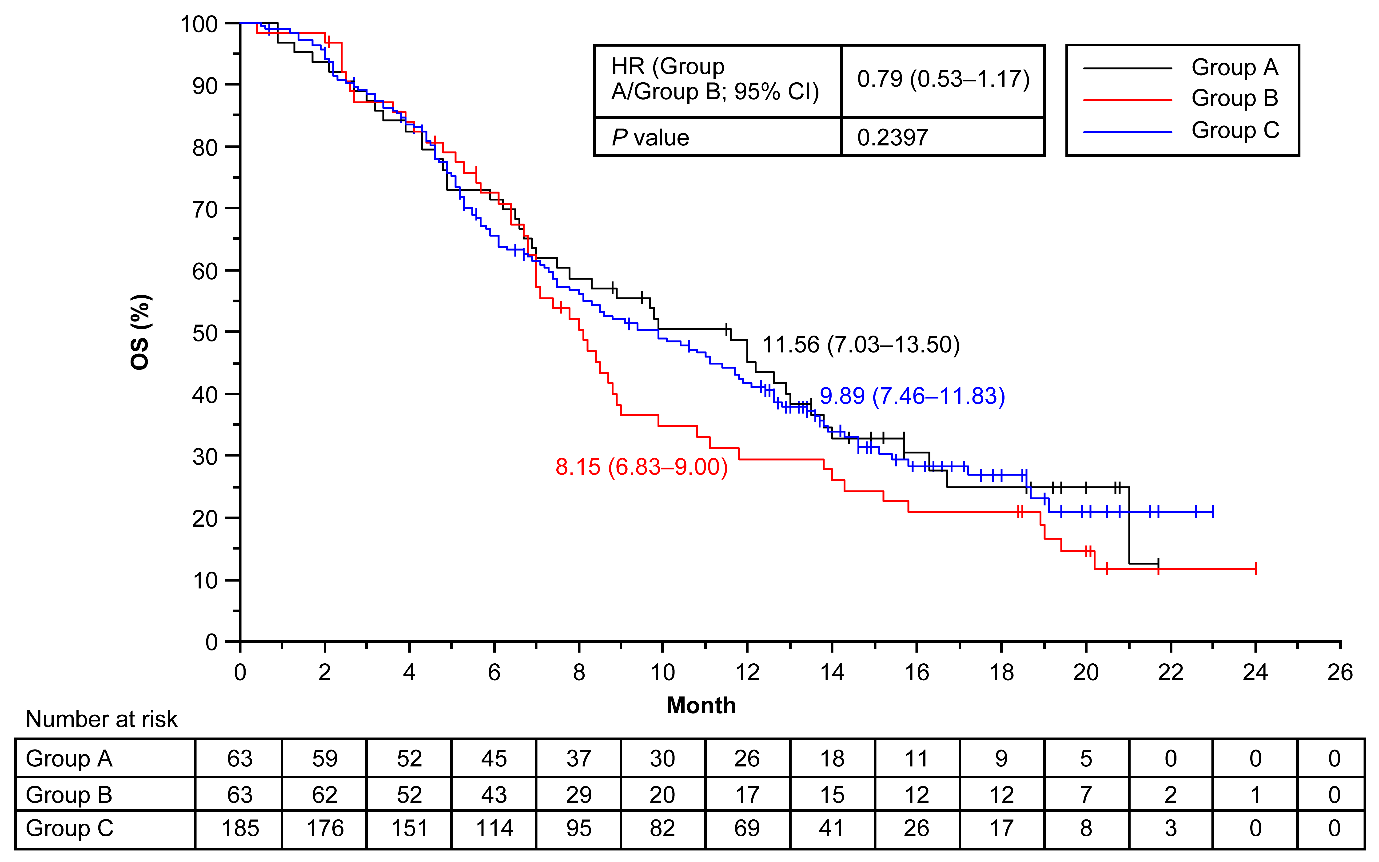


The numbers alongside the curves indicate the median OS and 95% CI.

Group A, patients who received their first dose of T-DXd treatment within 2 months after the final dose of nivolumab; Group B, patients who received their first dose of T-DXd treatment more than 2 months after the final dose of nivolumab; Group C, patients who did not receive nivolumab prior to T-DXd treatment.

CI, confidence interval; HR, hazard ratio; ICI, immune checkpoint inhibitor; OS, overall survival; T-DXd, trastuzumab deruxtecan.

**Figure S2** Relative OS across subgroups stratified by timing of ICI therapy prior to T-DXd administration


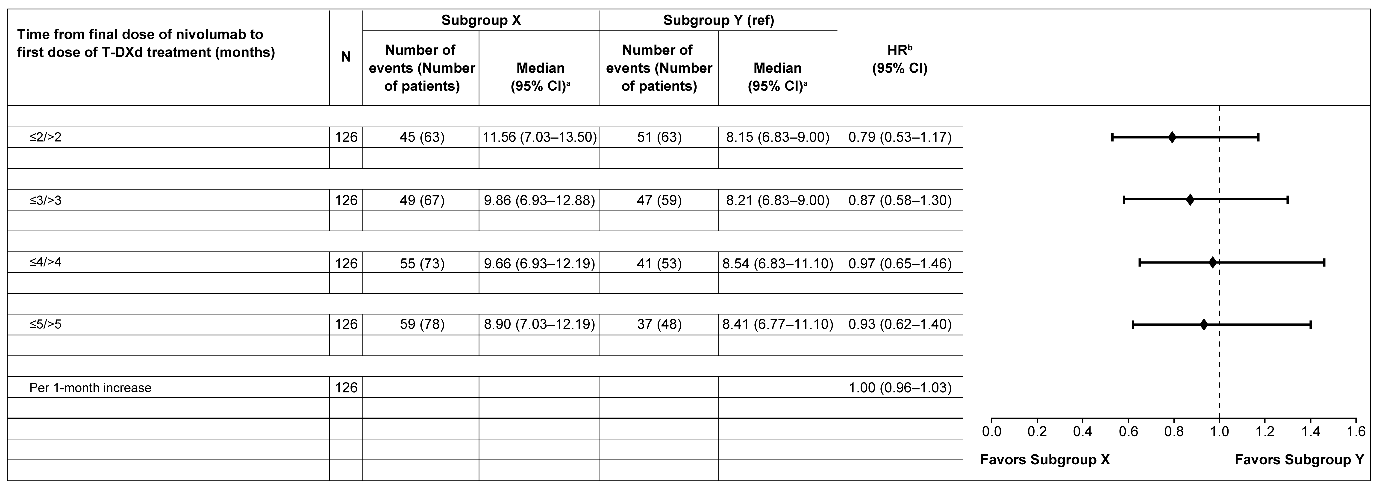


^a^Using the Brookmeyer and Crowley method.

^b^Using the Cox proportional hazards model.

Subgroup X, patients who received their first dose of T-DXd within a shorter time interval (≤2, ≤3, ≤4, or ≤5 months) following the final dose of nivolumab; Subgroup Y, patients who initiated T-DXd after a longer time interval (>2, >3, >4, or >5 months) following the final nivolumab dose.

CI, confidence interval; HR, hazard ratio; OS, overall survival; ref, reference; T-DXd, trastuzumab deruxtecan.

**Table S1** Subgroup analysis of therapies administered after T-DXd treatment

|  | Group A  n = 63 | Group B  n = 63 | Group C  n = 185 |
| --- | --- | --- | --- |
| Treatment immediately after T-DXd treatment |  |  |  |
| Yes | 34 (54.0) | 30 (47.6) | 130 (70.3) |
| No | 29 (46.0) | 33 (52.4) | 55 (29.7) |
| Type of treatment^a^ |  |  |  |
| Chemotherapy | 32 (50.8) | 25 (39.7) | 122 (65.9) |
| Radiation therapy | 1 (1.6) | 1 (1.6) | 3 (1.6) |
| BSC | 1 (1.6) | 4 (6.3) | 5 (2.7) |
| Others | 0 (0.0) | 0 (0.0) | 1 (0.5) |
| Therapies^a^ |  |  |  |
| Immune checkpoint inhibitor | 1 (1.6) | 2 (3.2) | 96 (51.9) |
| Nivolumab | 1 (1.6) | 2 (3.2) | 96 (51.9) |
| Pembrolizumab | 0 (0.0) | 0 (0.0) | 0 (0.0) |
| Ramucirumab | 2 (3.2) | 3 (4.8) | 8 (4.3) |
| Taxane | 2 (3.2) | 4 (6.3) | 3 (1.6) |
| Paclitaxel | 1 (1.6) | 0 (0.0) | 1 (0.5) |
| Nab-paclitaxel | 0 (0.0) | 1 (1.6) | 2 (1.1) |
| Docetaxel | 1 (1.6) | 3 (4.8) | 0 (0.0) |
| Platinum | 2 (3.2) | 4 (6.3) | 2 (1.1) |
| Cisplatin | 0 (0.0) | 0 (0.0) | 0 (0.0) |
| Oxaliplatin | 2 (3.2) | 4 (6.3) | 2 (1.1) |
| Irinotecan hydrochloride hydrate | 10 (15.9) | 1 (1.6) | 6 (3.2) |
| Intravenous drip infusion 40 mg, 100 mg | 7 (11.1) | 0 (0.0) | 6 (3.2) |
| Other | 3 (4.8) | 1 (1.6) | 0 (0.0) |
| Pyrimidine fluoride | 2 (3.2) | 7 (11.1) | 2 (1.1) |
| Capecitabine | 0 (0.0) | 2 (3.2) | 0 (0.0) |
| Tegafur-uracil | 0 (0.0) | 0 (0.0) | 0 (0.0) |
| Tegafur-gimeracil-oteracil potassium | 1 (1.6) | 3 (4.8) | 1 (0.5) |
| Fluorouracil | 1 (1.6) | 2 (3.2) | 1 (0.5) |
| Calcium levofolinate | 1 (1.6) | 2 (3.2) | 1 (0.5) |
| Trastuzumab | 1 (1.6) | 1 (1.6) | 2 (1.1) |
| Trastuzumab BS intravenous drip infusion 60 mg, 150 mg | 0 (0.0) | 1 (1.6) | 1 (0.5) |
| Other | 1 (1.6) | 0 (0.0) | 1 (0.5) |
| Other | 17 (27.0) | 11 (17.5) | 14 (7.6) |
| TAS-102 | 16 (25.4) | 11 (17.5) | 13 (7.0) |
| Epirubicin | 0 (0.0) | 0 (0.0) | 0 (0.0) |
| Investigational drugs | 1 (1.6) | 0 (0.0) | 0 (0.0) |
| Others | 0 (0.0) | 0 (0.0) | 1 (0.5) |
| T-DXd (genetical recombination) | 0 (0.0) | 0 (0.0) | 1 (0.5) |

Data are presented as n (%) unless otherwise specified.

^a^Multiple selections were allowed

Group A, patients who received their first dose of T-DXd treatment within 2 months after the final dose of nivolumab; Group B, patients who received their first dose of T-DXd treatment more than 2 months after the final dose of nivolumab; Group C, patients who did not receive nivolumab prior to T-DXd treatment.

BS, biosimilar; BSC, best supportive care; TAS-102, trifluridine, tipiracil hydrochloride; T-DXd, trastuzumab deruxtecan.

**Table S2** Univariate and multivariate analysis of rwPFS^a^

| **Factor** | **Category** | **Univariate analysis** | | | | **Multivariate analysis^b^** | |
| --- | --- | --- | --- | --- | --- | --- | --- |
|  |  | **Number of events (number of patients)** | **Median  (95% CI)^c^** | **HR^d^ (95% CI)** | ***P* value^d^** | **HR^d^ (95% CI)** | ***P* value^d^** |
| **Sex** | Male | 199 (232) | 4.57 (3.94–5.13) | 0.99 (0.75–1.31) | 0.9402 |  |  |
|  | Female | 66 (75) | 4.50 (3.42–5.22) | Reference |  |  |  |
| **Age (years)** | <65 | 85 (95) | 4.17 (3.29–4.86) | 1.13 (0.87–1.46) | 0.3519 |  |  |
|  | ≥65 | 180 (212) | 4.73 (4.14–5.36) | Reference |  |  |  |
| **ECOG PS^e^** | 0 | 108 (131) | 5.32 (4.50–6.34) | 0.72 (0.56–0.92) | 0.0077 | 0.85 (0.64–1.12) | 0.2404 |
|  | ≥1 | 155 (174) | 3.88 (3.29–4.57) | Reference |  | Reference |  |
| **HER2 status (IHC and ISH): Before T-DXd treatment^f^** | IHC 3+ | 180 (213) | 4.86 (4.24–5.49) | 0.68 (0.52–0.89) | 0.0048 | 0.62 (0.47–0.83) | 0.0012 |
|  | IHC 2+ and ISH+ | 77 (84) | 3.48 (2.40–4.17) | Reference |  | Reference |  |
| **Site of primary lesions** | Stomach | 223 (261) | 4.63 (3.94–5.22) | 0.83 (0.59–1.15) | 0.2613 |  |  |
|  | GEJ | 42 (46) | 4.27 (3.48–5.09) | Reference |  |  |  |
| **Any surgeries for primary lesions** | Yes | 87 (103) | 5.78 (4.63–6.93) | 0.70 (0.54–0.90) | 0.0064 | 0.83 (0.62–1.11) | 0.1991 |
|  | None | 178 (204) | 4.17 (3.45–4.57) | Reference |  | Reference |  |
| **Histological type of primary lesions^g^** | Intestinal | 143 (169) | 5.22 (4.53–6.24) | 0.62 (0.46–0.82) | 0.0010 | 0.62 (0.45–0.83) | 0.0018 |
|  | Diffuse | 70 (78) | 3.29 (2.27–4.17) | Reference |  | Reference |  |
| **Number of metastatic organs^h^** | 1 | 97 (117) | 5.06 (4.04–5.78) | 0.86 (0.67–1.10) | 0.2273 |  |  |
|  | ≥2 | 167 (189) | 4.21 (3.61–4.86) | Reference |  |  |  |
| **Metastasis site: Liver** | None | 129 (150) | 4.63 (3.71–5.13) | 1.05 (0.82–1.33) | 0.7033 |  |  |
|  | Yes | 136 (157) | 4.44 (3.94–5.36) | Reference |  |  |  |
| **Metastasis site: Peritoneum** | None | 166 (195) | 5.06 (4.37–5.75) | 0.76 (0.60–0.98) | 0.0352 | 0.96 (0.71–1.29) | 0.7814 |
|  | Yes | 99 (112) | 3.71 (2.96–4.57) | Reference |  | Reference |  |
| **Ascites^i^** | None | 142 (171) | 5.32 (4.57–6.24) | 0.65 (0.51–0.83) | 0.0005 | 0.84 (0.62–1.14) | 0.2609 |
|  | Yes | 120 (132) | 3.42 (2.69–4.21) | Reference |  | Reference |  |
| **mGPS^j^** | 0 and 1 | 192 (225) | 5.06 (4.44–5.49) | 0.64 (0.48–0.84) | 0.0017 | 0.67 (0.49–0.90) | 0.0079 |
|  | 2 | 66 (74) | 3.02 (2.20–3.94) | Reference |  | Reference |  |
| **LDH (U/L)^h^** | ≤222 | 141 (169) | 4.86 (4.04–5.55) | 0.85 (0.67–1.09) | 0.1941 |  |  |
|  | >222 | 123 (137) | 4.17 (3.42–4.63) | Reference |  |  |  |
| **ALP (U/L)^k^** | <ULN | 108 (127) | 5.09 (4.50–6.01) | 0.85 (0.67–1.09) | 0.2105 |  |  |
|  | ≥ULN | 151 (174) | 4.04 (3.48–4.73) | Reference |  |  |  |
| **Number of lines of previous therapy** | 2 | 133 (156) | 4.21 (3.68–4.86) | 1.07 (0.84–1.37) | 0.5666 |  |  |
|  | ≥3 | 132 (151) | 4.99 (3.94–5.68) | Reference |  |  |  |
| **CrCl (mL/min)** | ≥Median | 135 (154) | 4.34 (3.84–5.13) | 1.08 (0.84–1.37) | 0.5552 |  |  |
|  | <Median | 130 (153) | 4.63 (3.94–5.36) | Reference |  |  |  |
| **Hb (g/dL)** | ≥Median | 130 (155) | 4.86 (4.21–5.72) | 0.84 (0.66–1.07) | 0.1582 |  |  |
|  | <Median | 135 (152) | 3.94 (3.29–5.06) | Reference |  |  |  |
| **NLR** | <Median | 128 (154) | 4.86 (3.98–5.75) | 0.80 (0.63–1.01) | 0.0650 |  |  |
|  | ≥Median | 137 (153) | 4.21 (3.61–4.93) | Reference |  |  |  |
| **Time from final dose of nivolumab to first dose of T-DXd treatment (months)^h^** | ≤2 | 50 (63) | 6.93 (4.57–7.79) | 0.62 (0.45–0.86) | 0.0039 | 0.63 (0.44–0.89) | 0.0081 |
|  | >2 | 58 (63) | 4.63 (3.75–5.75) | 1.01 (0.75–1.37) | 0.9255 | 0.97 (0.70–1.35) | 0.8595 |
|  | None | 156 (180) | 4.17 (3.71–4.57) | Reference |  | Reference |  |
| **Duration of trastuzumab treatment before T-DXd treatment (months)^l^** | ≥Median (≥6.5) | 120 (142) | 5.36 (4.44–6.47) | 0.70 (0.55–0.91) | 0.0062 | 0.75 (0.57–0.97) | 0.0298 |
|  | <Median (<6.5) | 125 (141) | 3.71 (3.02–4.21) | Reference |  | Reference |  |

ALP, alkaline phosphatase; CI, confidence interval; CrCl, creatinine clearance; ECOG PS, Eastern Cooperative Oncology Group performance status; GEJ, gastroesophageal junction; Hb, hemoglobin; HER2, human epidermal growth factor receptor 2; HR, hazard ratio; IHC, immunohistochemistry; ISH, *in situ* hybridization; LDH, lactate dehydrogenase; mGPS, modified Glasgow Prognostic Score; NLR, neutrophil to lymphocyte ratio; rwPFS, real-world progression-free survival; T-DXd, trastuzumab deruxtecan; ULN, upper limit of normal.

^a^N = 307 unless otherwise specified.

^b^Factors with *P* < .05 in the univariate analysis were included in the multivariate analysis.

^c^Brookmeyer and Crowley method.

^d^Using the Cox proportional hazards model.

^e^n = 305.

^f^n = 297.

^g^n = 247.

^h^n = 306.

^i^n = 303.

^j^n = 299.

^k^n = 301.

^l^n = 283.

**Table S3** Univariate and multivariate analysis of OS^a^

| **Factor** | **Category** | **Univariate analysis** | | | | **Multivariate analysis^b^** | |
| --- | --- | --- | --- | --- | --- | --- | --- |
|  |  | **Number of events (number of patients)** | **Percentage  (95% CI)^c^** | **HR^d^ (95% CI)** | ***P* value^d^** | **HR^d^ (95% CI)** | ***P* value^d^** |
| **Sex** | Male | 161 (232) | 9.13 (8.15–11.56) | 0.80 (0.59–1.08) | 0.1462 |  |  |
|  | Female | 57 (75) | 7.82 (5.88–11.70) | Reference |  |  |  |
| **Age, years** | <65 | 71 (95) | 7.82 (5.91–11.93) | 1.12 (0.84–1.49) | 0.4361 |  |  |
|  | ≥65 | 147 (212) | 9.66 (8.21–11.43) | Reference |  |  |  |
| **ECOG PS^e^** | 0 | 79 (131) | 12.85 (10.05–15.70) | 0.52 (0.39–0.69) | <0.0001 | 0.61 (0.44–0.83) | 0.0019 |
|  | ≥1 | 139 (174) | 7.23 (6.24–8.54) | Reference |  | Reference |  |
| **HER2 status: Before T-DXd treatment^f^** | IHC 3+ | 148 (213) | 10.45 (8.15–11.93) | 0.68 (0.51–0.91) | 0.0089 | 0.54 (0.39–0.74) | 0.0001 |
|  | IHC 2+ and ISH+ | 67 (84) | 7.43 (5.95–8.74) | Reference |  | Reference |  |
| **Site of primary lesions** | Stomach | 181 (261) | 8.90 (7.82–10.84) | 0.85 (0.60–1.21) | 0.3736 |  |  |
|  | GEJ | 37 (46) | 8.67 (4.93–13.70) | Reference |  |  |  |
| **Any surgeries for primary lesions** | Yes | 69 (103) | 12.35 (9.00–14.29) | 0.69 (0.52–0.92) | 0.0125 | 1.04 (0.75–1.44) | 0.8358 |
|  | None | 149 (204) | 7.82 (6.83–9.36) | Reference |  | Reference |  |
| **Histological type of primary lesions^g^** | Intestinal | 111 (169) | 11.14 (8.90–12.88) | 0.52 (0.38–0.70) | <0.0001 | 0.52 (0.38–0.72) | <0.0001 |
|  | Diffuse | 65 (78) | 6.14 (5.13–7.36) | Reference |  | Reference |  |
| **Number of metastatic organs^h^** | 1 | 73 (117) | 12.55 (10.81–14.55) | 0.58 (0.43–0.77) | 0.0002 | 0.70 (0.51–0.97) | 0.0302 |
|  | ≥2 | 144 (189) | 7.52 (6.37–8.77) | Reference |  | Reference |  |
| **Metastasis site: Liver** | None | 110 (150) | 9.36 (7.52–11.79) | 1.07 (0.82–1.39) | 0.6367 |  |  |
|  | Yes | 108 (157) | 8.54 (7.26–11.43) | Reference |  |  |  |
| **Metastasis site: Peritoneum** | None | 131 (195) | 11.14 (8.90–13.40) | 0.62 (0.47–0.81) | 0.0005 | 0.85 (0.61–1.19) | 0.3433 |
|  | Yes | 87 (112) | 7.03 (6.14–8.31) | Reference |  | Reference |  |
| **Ascites^i^** | None | 110 (171) | 12.19 (9.89–13.96) | 0.52 (0.39–0.68) | <0.0001 | 0.75 (0.53–1.04) | 0.0863 |
|  | Yes | 106 (132) | 6.74 (5.68–7.46) | Reference |  | Reference |  |
| **mGPS^j^** | 0 and 1 | 153 (225) | 11.07 (9.13–12.65) | 0.46 (0.34–0.62) | <0.0001 | 0.65 (0.46–0.90) | 0.0089 |
|  | 2 | 63 (74) | 5.62 (4.40–6.93) | Reference |  | Reference |  |
| **LDH (U/L)^h^** | ≤222 | 116 (169) | 11.04 (8.74–12.55) | 0.68 (0.52–0.88) | 0.0043 | 0.85 (0.63–1.14) | 0.2820 |
|  | >222 | 102 (137) | 7.46 (6.28–8.77) | Reference |  | Reference |  |
| **ALP (U/L)^k^** | <ULN | 88 (127) | 11.07 (8.90–13.50) | 0.79 (0.60–1.04) | 0.0898 |  |  |
|  | ≥ULN | 126 (174) | 7.82 (6.74–9.36) | Reference |  |  |  |
| **Number of lines of previous therapy** | 2 | 107 (156) | 9.13 (7.43–11.70) | 0.95 (0.72–1.23) | 0.6834 |  |  |
|  | ≥3 | 111 (151) | 8.90 (7.26–11.56) | Reference |  |  |  |
| **CrCl (mL/min)** | ≥Median | 111 (154) | 8.90 (7.43–11.07) | 1.05 (0.81–1.37) | 0.7124 |  |  |
|  | <Median | 107 (153) | 8.90 (7.23–12.62) | Reference |  |  |  |
| **Hb (g/dL)** | ≥Median | 101 (155) | 11.73 (9.00–13.77) | 0.74 (0.57–0.97) | 0.0273 | 1.00 (0.74–1.34) | 0.9799 |
|  | <Median | 117 (152) | 7.95 (6.60–8.90) | Reference |  | Reference |  |
| **NLR** | <Median | 97 (154) | 11.73 (8.74–13.83) | 0.60 (0.46–0.79) | 0.0002 | 0.72 (0.54–0.96) | 0.0270 |
|  | ≥Median | 121 (153) | 7.26 (5.85–8.90) | Reference |  | Reference |  |
| **Time from final dose of nivolumab to first dose of T-DXd treatment (month)^h^** | ≤2 | 45 (63) | 11.56 (7.03–13.50) | 0.94 (0.67–1.32) | 0.7216 |  |  |
|  | >2 | 51 (63) | 8.15 (6.83–9.00) | 1.19 (0.86–1.65) | 0.3029 |  |  |
|  | None | 121 (180) | 9.40 (7.46–11.73) | Reference |  |  |  |
| **Duration of trastuzumab treatment before T-DXd treatment (months)^l^** | ≥Median  (≥6.5) | 94 (142) | 12.19 (9.00–13.77) | 0.64 (0.48–0.84) | 0.0015 | 0.65 (0.48–0.87) | 0.0035 |
|  | <Median (<6.5) | 107 (141) | 7.13 (6.24–8.80) | Reference |  | Reference |  |

ALP, alkaline phosphatase; CI, confidence interval; CrCl, creatinine clearance; ECOG PS, Eastern Cooperative Oncology Group performance status; GEJ, gastroesophageal junction; Hb, hemoglobin; HER2, human epidermal growth factor receptor 2; HR, hazard ratio; IHC, immunohistochemistry; ISH, in situ hybridization; LDH, lactate dehydrogenase; mGPS, modified Glasgow Prognostic Score; NLR, neutrophil to lymphocyte ratio; OS, overall survival; T-DXd, trastuzumab deruxtecan; U/L, units per liter; ULN, upper limit of normal.

^a^N = 307 unless otherwise specified.

^b^Factors with *P* < .05 in the univariate analysis were included in the multivariate analysis.

^c^Brookmeyer and Crowley method.

^d^Using the Cox proportional hazards model.

^e^n = 305.

^f^n = 297.

^g^n = 247.

^h^n = 306.

^i^n = 303.

^j^n = 299.

^k^n = 301.

^l^n = 283.

**Table S4** Univariate and multivariate analysis of ORR^a^

| **Factor** | **Category** | **Univariate analysis** | | | | **Multivariate analysis^b^** | |
| --- | --- | --- | --- | --- | --- | --- | --- |
|  |  | **Number of events (number of patients)** | **Percentage  (95% CI)^c^** | **OR^d^ (95% CI)** | ***P*-value^d^** | **OR^d^ (95% CI)** | ***P*-value^d^** |
| **Sex** | Male | 70 (169) | 41.4 (33.9–49.2) | 0.85 (0.46–1.57) | 0.5992 |  |  |
|  | Female | 25 (55) | 45.5 (32.0–59.4) | Reference |  |  |  |
| **Age, years** | <65 | 31 (67) | 46.3 (34.0–58.9) | 1.25 (0.70–2.23) | 0.4457 |  |  |
|  | ≥65 | 64 (157) | 40.8 (33.0–48.9) | Reference |  |  |  |
| **ECOG PS^e^** | 0 | 42 (97) | 43.3 (33.3–53.7) | 1.04 (0.61–1.77) | 0.8932 |  |  |
|  | ≥1 | 53 (125) | 42.4 (33.6–51.6) | Reference |  |  |  |
| **HER2 status: Before T-DXd treatment^f^** | IHC 3+ | 70 (147) | 47.6 (39.3–56.0) | 1.86 (1.02–3.40) | 0.0441 | 1.62 (0.86–3.07) | 0.1354 |
|  | IHC 2+ and ISH+ | 22 (67) | 32.8 (21.8–45.4) | Reference |  | Reference |  |
| **Site of primary lesions** | Stomach | 78 (193) | 40.4 (33.4–47.7) | 0.56 (0.26–1.20) | 0.1349 |  |  |
|  | GEJ | 17 (31) | 54.8 (36.0–72.7) | Reference |  |  |  |
| **Any surgeries for primary lesions** | Yes | 40 (82) | 48.8 (37.6–60.1) | 1.51 (0.87–2.61) | 0.1435 |  |  |
|  | None | 55 (142) | 38.7 (30.7–47.3) | Reference |  |  |  |
| **Histological type of primary lesions^g^** | Intestinal | 65 (132) | 49.2 (40.4–58.1) | 1.58 (0.81–3.08) | 0.1760 |  |  |
|  | Diffuse | 19 (50) | 38.0 (24.7–52.8) | Reference |  |  |  |
| **Number of metastatic organs** | 1 | 32 (73) | 43.8 (32.2–55.9) | 1.09 (0.62–1.92) | 0.7637 |  |  |
|  | ≥2 | 63 (151) | 41.7 (33.8–50.0) | Reference |  |  |  |
| **Metastasis site: Liver** | None | 34 (84) | 40.5 (29.9–51.7) | 0.88 (0.51–1.52) | 0.6500 |  |  |
|  | Yes | 61 (140) | 43.6 (35.2–52.2) | Reference |  |  |  |
| **Metastasis site: Peritoneum** | None | 72 (161) | 44.7 (36.9–52.7) | 1.41 (0.77–2.56) | 0.2645 |  |  |
|  | Yes | 23 (63) | 36.5 (24.7–49.6) | Reference |  |  |  |
| **Ascites^h^** | None | 63 (137) | 46.0 (37.4–54.7) | 1.43 (0.82–2.49) | 0.2102 |  |  |
|  | Yes | 31 (83) | 37.3 (27.0–48.7) | Reference |  |  |  |
| **mGPS^i^** | 0 and 1 | 76 (159) | 47.8 (39.8–55.9) | 1.98 (1.05–3.76) | 0.0357 | 1.91 (0.98–3.71) | 0.0575 |
|  | 2 | 18 (57) | 31.6 (19.9–45.2) | Reference |  | Reference |  |
| **LDH (U/L)^j^** | ≤222 | 47 (115) | 40.9 (31.8–50.4) | 0.90 (0.53–1.53) | 0.6889 |  |  |
|  | >222 | 47 (108) | 43.5 (34.0–53.4) | Reference |  |  |  |
| **ALP (U/L)^h^** | <ULN | 43 (94) | 45.7 (35.4–56.3) | 1.24 (0.72–2.13) | 0.4348 |  |  |
|  | ≥ULN | 51 (126) | 40.5 (31.8–49.6) | Reference |  |  |  |
| **Number of lines of previous therapy** | 2 | 47 (107) | 43.9 (34.3–53.9) | 1.13 (0.66–1.91) | 0.6610 |  |  |
|  | ≥3 | 48 (117) | 41.0 (32.0–50.5) | Reference |  |  |  |
| **CrCl (mL/min)** | ≥Median | 54 (115) | 47.0 (37.6–56.5) | 1.47 (0.86–2.50) | 0.1580 |  |  |
|  | <Median | 41 (109) | 37.6 (28.5–47.4) | Reference |  |  |  |
| **Hb (g/dL)** | ≥Median | 54 (114) | 47.4 (37.9–56.9) | 1.51 (0.89–2.58) | 0.1271 |  |  |
|  | <Median | 41 (110) | 37.3 (28.2–47.0) | Reference |  |  |  |
| **NLR** | <Median | 52 (113) | 46.0 (36.6–55.6) | 1.35 (0.79–2.29) | 0.2709 |  |  |
|  | ≥Median | 43 (111) | 38.7 (29.6–48.5) | Reference |  |  |  |
| **Time from final dose of nivolumab to first dose of T-DXd treatment (month)^j^** | ≤2 | 28 (51) | 54.9 (40.3–68.9) | 1.65 (0.85–3.19) | 0.1382 |  |  |
|  | >2 | 16 (52) | 30.8 (18.7–45.1) | 0.60 (0.30–1.20) | 0.1493 |  |  |
|  | None | 51 (120) | 42.5 (33.5–51.9) | Reference |  |  |  |
| **Duration of trastuzumab treatment before T-DXd treatment (months)^k^** | ≥Median  (≥6.5) | 50 (97) | 51.5 (41.2–61.8) | 2.25 (1.28–3.96) | 0.0050 | 2.02 (1.13–3.63) | 0.0181 |
|  | <Median (<6.5) | 35 (109) | 32.1 (23.5–41.7) | Reference |  | Reference |  |

ALP, alkaline phosphatase; CI, confidence interval; CrCl, creatinine clearance; ECOG PS, Eastern Cooperative Oncology Group performance status; GEJ, gastroesophageal junction; Hb, hemoglobin; HER2, human epidermal growth factor receptor 2; IHC, immunohistochemistry; ISH, *in situ* hybridization; LDH, lactate dehydrogenase; mGPS, modified Glasgow Prognostic Score; NLR, neutrophil to lymphocyte ratio; OR, odds ratio; ORR, objective response rate; T-DXd, trastuzumab deruxtecan; U/L, units per liter; ULN, upper limit of normal.

^a^N = 224 unless otherwise specified.

^b^Factors with *P* < .05 in the univariate analysis were included in the multivariate analysis.

^c^Clopper-Pearson method.

^d^Using the logistic regression model.

^e^n = 222.

^f^n = 214.

^g^n = 182.

^h^n = 220.

^i^n = 216.

^j^n = 223.

^k^n = 206.
